# Why do consumers choose private over public health services? Reflective accounts of health providers in Vietnam

**DOI:** 10.1186/s12913-023-09892-9

**Published:** 2023-08-23

**Authors:** Mai P. Nguyen, Amina Tariq, Reece Hinchcliff, Michael P. Dunne

**Affiliations:** 1 Department of Medical Services Administration, Ministry of Health, Hanoi, Vietnam; 2 School of Public Health & Social Work, Faculty of Health, Queensland University of Technology, Brisbane, QLD Australia; 3 Australian Centre for Health Services Innovation (AusHSI) and Centre for Healthcare Transformation, School of Public Health & Social Work, Queensland University of Technology, Brisbane, QLD Australia; 4 School of Applied Psychology, Griffith Health Group, Griffith University, Queensland, Australia; 5 Institute for Community Health Research, Hue University, Hue, Vietnam; 6 Australian Centre for Health Law Research, Queensland University of Technology, Queensland, Australia

**Keywords:** Service utilisation, Private facilities, Public health system, Social interaction, Vietnam

## Abstract

**Background:**

In Vietnam and many developing countries, private healthcare is increasingly being leveraged by governments to complement public services and increase health service access and utilisation. Extensive understanding of patterns of utilisation of private over public health services, and the rationale for such consumer decisions, is important to ensure and promote safe, affordable and patient-centred care in the two sectors. Few studies within the Southeast Asian Region have explored how private and public providers interact (via social networks, marketing, and direct contact) with consumers to affect their service choices. This study investigates providers’ views on social factors associated with the use of private over public health services in Vietnam.

**Method:**

A thematic analysis was undertaken of 30 semi-structured interviews with experienced health system stakeholders from the Vietnam national assembly, government ministries, private health associations, health economic association, as well as public and private hospitals and clinics.

**Results:**

Multiple social factors were found to influence the choice of private over public services, including word-of-mouth, the patient-doctor relationship and relationships between healthcare providers, healthcare staff attitudes and behaviour, and marketing. While private providers maximise their use of these social factors, most public providers seem to ignore or show only limited interest in using marketing and other forms of social interaction to improve services to meet patients’ needs, especially those needs beyond strictly medical intervention. However, private providers faced their own particular challenges related to over-advertisement, over-servicing, excessive focus on patients’ demands rather than medical needs, as well as the significant technical requirements for quality and safety.

**Conclusions:**

This study has important implications for policy and practice in Vietnam. First, public providers must embrace social interaction with consumers as an effective strategy to improve their service quality. Second, appropriate regulations of private providers are required to protect patients from unnecessary treatments, costs and potential harm. Finally, the insights from this study have direct relevance to many developing countries facing a similar challenge of appropriately managing the growth of the private health sector.

**Supplementary Information:**

The online version contains supplementary material available at 10.1186/s12913-023-09892-9.

## Background

The health system of Vietnam comprises both public and private providers, of which public providers are dominant, resembling many other Southeast Asian countries [1–3]. According to data published by the Vietnam Ministry of Health in 2020, there were 13,316 public health facilities, including 1,235 hospitals, 11,810 health stations (or public clinics), and health units. Private health facilities include 228 hospitals and only 6% of total nationwide hospital beds [4].

The public sector currently fails to meet many of the diverse healthcare needs of the Vietnamese population. Public facilities, especially large public hospitals, face challenges of overcrowding, long waiting times, complicated administration procedures, unempathetic staff, corruption, and shortages of high-tech and modern equipment [5]. Within this context, the private sector in Vietnam provides opportunities to complement the public sector and other government efforts to support social and economic development, and achieve universal health coverage [6].

The growth of participation by the private sector in health service provision requires appropriate regulations and controls to ensure high-quality, affordable, patient-centred care [6]. In addition, expansion of the role and involvement of private health services should not widen disparities in health access and quality, or substantially increase the overall cost of the healthcare system. Exploration of these issues necessitates an improved understanding of patterns of utilisation of private over public health services, and the rationale for such consumer decisions.

Global knowledge and practice have shown the complex and diverse impacts of social interaction on patients’ choice of providers [7–9]. Social interaction refers to the interdependencies among patients, between patients and healthcare providers, and among social and corporate networks of health service providers [10]. In some contexts, consumers’ interaction with individuals and networks can be the most influential factor determining the choice of private or public health services [11].

Many previous studies focused on consumers’ characteristics to understand why and how an individual decided to choose private instead of public health services. Age, income and education have been identified as key individual factors affecting consumers’ choice of private over public health services in Vietnam [12, 13]. However, there is still limited understanding of how private and public providers interact (via social networks, marketing, and direct contact) with consumers to affect their choices. This study aimed to address this gap by investigating providers’ views on how these factors affect the use of private over public health services in Vietnam.

## Methods

Data was collected through semi-structured interviews with key informants of the Vietnamese health system who were purposely selected. Participants were identified and located through the first author’s professional networks (as a civil servant of the Ministry of Health) and snowballing (one respondent suggesting another). The final sample size was determined at the point of saturation, when the perspectives from participants became repetitive, meaning that adding more participants to the study would be unlikely to obtain additional, useful information. Our study included only informants from middle-level to senior professionals who had 10 or more years of experience in management/leadership positions in the health sector.

Thirty experienced key informants were recruited, including one policymaker from the Vietnam National Assembly, six informants from government ministries, four respondents from government bodies that regulate healthcare, two senior managers from private health associations, one expert from the Health Economic Association, three managers/directors of public hospitals and clinics, nine directors of private hospitals and clinics, two researchers from national research institutes, and two respondents from international organisations in Vietnam.

All interviews were conducted by the first author in Hanoi, Hue, and Ho Chi Minh City in the Northern, Central and Southern regions of Vietnam, in 2019. They took from 23 to 90 min and were held in private locations where confidentiality was assured. The range of interview length was due to extraneous circumstances, including the availability of participants and their depth of insight offered regarding the research topics. The guiding questions were, (1) why do consumers choose, or elect not to choose, private instead of public health providers (services)?, (2) what are possible types of cooperation, services that could be encouraged to develop private healthcare towards national health goals?, (3) What are the barriers and facilitators in terms of regulation for the development of private health sector?. Participants were also encouraged to propose recommendations on policy formulation and/or revision to foster the development of private service usage and discuss new emerging health system issues and challenges in Vietnam. Twenty-eight of the thirty interviews were recorded electronically as permitted by informants, while two interviews were recorded via note-taking by the first author.

Data were analyzed inductively so that themes emerged directly from the data [14]. The initial codes were generated by reading interview transcripts and observation notes. All coded nodes were then categorized and organized in a meaningful relation and interpretable way. Finally, themes were developed based on groups of codes. NVivo software package version 12 was used to assist in organising, analysing, and revealing insights into the qualitative dataset. The confirmability, validity, and reliability of the findings were iteratively checked by the research team throughout the entire process of analysis, to ensure reliable research outcomes.

The first author interviewed and recorded all interviews using digital recorders and took notes where possible. The transcriptions were completed by a transcriber, then double-checked with the original audio records in Vietnamese by the first author. After open coding, the codes were translated from Vietnamese into English, preserving the original meanings. Members of the research team then edited the translated versions to be readable and understandable in English. They also cross-checked the codes and themes, then referred to the literature to support findings.

## Results

The participants were all mid- to senior-level and had ten or more years of experience in management/leadership positions in the health sector. All interviewed directors of private hospitals were medical doctors who had been practising in public hospitals for more than 15 years. Five of the six interviewed private clinic owners were specialist physicians and had been working in public hospitals or health centres. Only one private clinic owner worked alone (without working dually in a public facility). Details of respondents are shown in Appendix 1.

Five main themes related to the influence of social interaction on individual choice of private over public services were identified by interviewees, and are summarised in Table 1.

**Table 1:** Social factors influencing the choice of private over public health services

Themes	Definition
1. Word-of-mouth	Consumers share information about medical conditions and treatment experiences with peers and recommend health providers
2. Relationship	How relationships among patients and physicians change the decision whether to use private over public services
3. Staff behaviour and attitude	How medical, nursing, and supporting staff attitudes and behaviour affect the use of private over public services
4. Impacts of service reputation and brand	The influence of organisational reputation on the choice of services
5. Marketing	How providers promote their services to recruit patients

(*) the number of times a theme appears

### Importance of word-of-mouth

This theme reveals how sharing information through word-of-mouth about health services affects the use of private or public health services. Consumers frequently share their experiences with services with other consumers and this has a strong influence on the selection of healthcare services [15]. This “word-of-mouth communication” [16] is independent of providers and transmits spontaneously [17]. With the rapid expansion of social media platforms such as Facebook, Twitter, and YouTube, consumers increasingly go online to interact with their peers to share medical conditions and treatment experiences and to recommend health providers they think are best, and caution against services at which they had unsatisfactory experiences [18]. Consequently, word-of-mouth communication transmits more quickly than ever.

This study found that private providers in Vietnam had recognised the importance of word-of-mouth. It was regarded by participants as an effective advertising tool, directly targeting potential customers with key information, at a much lower cost than other types of marketing. As a result, institutions now strived to satisfy patients in many aspects so that they become “ambassadors” and disseminate positive views on service quality to their networks and the wider community, thus attracting more customers to private facilities. The following quotes highlight these issues; “Our hospital was just founded so we have quite many patients coming over through word-of-mouth” (Participant 11, director of private hospital_2), and “When patients are happy, they will recommend and bring other visitors to us” (Participant 13, private clinic owner_3).

Many dual-practice doctors were motivated to recruit patients who they first meet in public facilities. Overcrowding in public hospitals and under-staffing results was explained to result in minimal consultation time, per patient. To provide continuity of care for those who can afford to pay, some public doctors refer patients to clinics in which they work privately. The experience at public clinics provides an opportunity to promote their private services to patients’ families and social networks; “If a patient is well treated by doctors in the public hospital, he/she will advertise for those doctors, who will [then] have more patients in their private clinics” (Participant 12, private clinic owner_2).

### The influential role of relationships

This theme captured how relationships among patients and physicians, and those among physicians themselves, influence decisions regarding whether to use private over public services. It refers to a deeper mutual understanding between patients and doctors. The relation is based on already built-up communication and experience and trust among them, not just hearing from other peer consumers as in the theme “word-of-mouth”.

Patient-doctor relationships are established directly during patients’ visits to public facilities. Patients then know that a good public doctor often also runs a private clinic on evenings and weekends overtime and may decide to go there for shorter waiting times and longer consultations that are not feasible in public facilities. This was outlined by some private clinic owners:

In fact, I only see patients that I know, and they know me. These patients do not have time to go to public hospitals. So, after working hours, people come to my clinic to get more careful examinations. (Participant 3, private clinic owner_1)

I am still working in a public hospital and see patients there. But for patients who want to do a more private examination at a favourable time, they would go to my clinics outside office hours for a shorter waiting time, directly and fully communicate with me and [receive] a more personal treatment. (Participant 12, private clinic owner_ 2)

Moreover, many doctors refer patients to colleagues in their social and professional networks, as evident in the following quotes:

It takes a lot of time for private clinics to build a brand name. Thus, we must network and introduce ourselves. For example, I have a lot of colleagues and friends who work in the private sector in different specialties, and we introduce and recommend each other, from this specialty to that specialty, forming our own network system. (Participant 20, private clinic owner_5)

I worked for a long time in the public sector before I opened my own clinic, so I have a quite stable number of clients who have known me for a long time. (Participant 13, private clinic owner_3)

### Influence of staff attitudes and behaviour

Communication difficulties sometimes co-occur with corruption and bribery, where patients are informally but quite overtly expected to pay cash incentives to medical staff, nurses, pharmacists, administrators, and others, with the hope they receive better care and/or jump the long waiting queues. All interviewees acknowledged that the attitudes of staff in private facilities tend to be better than in public facilities, and this is attractive to patients, as outlined in the following quotes

Apart from the medical care itself, staff attitude and enthusiasm, etc., are more stable in the private sector than public one, and there are no harassment, nuisance, or “requirements” (a requirement for under-the-table payment) in private healthcare facilities. Communication between patients and medical staff is also more pleasant and friendly than in most public health facilities. (Participant 18, private clinic owner_4)

Why can private hospitals be attractive to patients? Besides the good quality of their services, the staff attitude is very good (without paying more money). (Participant 19, Congressman)

A patient comes to private providers because of their better staff attitude and communication with clients compared to those in public hospitals. (Participant 16, director of a major public hospital)

People prefer private facilities because of faster procedures and better staff attitude. (Participant 6, Health Economics Association expert)

Some participants emphasised that this problem was not just limited to physicians. The attitudes and behaviour of supporting staff also affect the choice of services because these staff often are the first point of contact at the facilities, and they are most likely to be the people who follow up directly with patients. This point was made during the following interview:

When the patient comes to [a] private clinic, the receptionist and supporting staff are very dedicated. They welcome the patient from arrival until they depart, so the patient is very appreciated and satisfied. When patients [were] newly arrived, they did not know where to do a medical examination, how to find and go to the needed places, the rooms… all of these issues were handled by enthusiastic staff who guided and brought them to the right place. (Participant 28, a public doctor working for a private clinic)

Interviewees noted that in public providers, many support staff have limited training in customer service. In a bid to compete with the private sector, public providers were viewed as being increasingly required to improve the attitudes of their staff, but it may take time to close the gap between the two sectors. These concepts are highlighted in the following quotes:

It is true that the attitude of staff is better in private hospitals than in public ones, but now the gap is narrowing because public hospitals have been [given] autonomy, so it is not possible to be rude to patients. But very soon, you will see that public hospitals have a welcoming and well-dressed staff, dedicated to talking to and guiding patients. However, private providers have a long tradition to invest in training a great reception team, and because of overcrowding in public hospitals, we might not do as well as they do. (Participant 25, Head of the Quality Assurance Department of a major public hospital)

One of the reasons why my private hospital attracts patients is the great working attitude of the whole team. At least, they all understand that their quite good salary comes from patients’ payments. (Participant 24, director of a private hospital)

The receptionists, the ushers and the nurse assistants, are very devoted, welcoming the patients from the time of arrival to the time they get out of the private facilities, so the patients feel satisfied. Many patients in public hospitals do not know where to go for their examinations, how to go to the consultation rooms, or how to find different places... In private providers, the extra staff are dedicated to guiding and bringing patients to the right place. (Participant 28, a retired public doctor working in a private clinic).

In summary, staff attitudes and the behaviour of clinicians and support staff are suboptimal in many public facilities, and this is a significant reason why patients choose private health services.

### The influence of service reputation and brand

This theme captured the influence of doctors’ reputations and organizational brands on consumers’ choice of services.

All participants acknowledged that doctors’ reputations and organizations’ brands, especially in relation to the perceived competence of the medical staff, are critical factors that determine the choice of either private or public services in Vietnam. Qualified medical staff are the key to a provider’s reputation. In general, it emerged throughout the interviews that public health facilities have had considerable community trust for decades in Vietnam, especially services in tertiary-level facilities (provincial and national level hospitals). Thus, patients often prefer them.

Participant 25, a director of a major public hospital said “Patients have been trusting the public sector much more than the private sector. That’s Vietnamese people’s long-lasting mindset”.

However, private providers seek to “borrow” or “grab” a reputation from the public sector and use it as an asset to attract more patients.

We contracted with some top professors, and doctors from major public hospitals to work overtime in our hospital to advise and/or conduct technically sophisticated surgeries and advanced technology (Participant 24, director of a private hospital 3)

Public doctors who wish to open private clinics often practice for many years to build up their expertise and reputation in the public sector before opening new clinics or join with private groups. Reputation is vital to attract an increasingly savvy population who are well aware of service quality and it has a great impact on consumers’ decision-making for price-sensitive decisions. A doctor who left a public hospital after more than 10 years of service said: “I think patients come to me because of my prestige because they already have faith. Their friends, parents, brothers and sisters have had their surgery performed here by me. They do not care much about the price” (Participant 12, a private clinic owner_2).

In summary, an organisation’s reputation is an important consideration when choosing private over public health services. While the public sector has traditionally been dominant in terms of high reputation and qualified doctors, the private sector is now attracting the top qualified doctors required to build up their reputation; thus, attracting more clients.

### Marketing

Given their dominant position in the Vietnamese healthcare sector and the over-abundance of people seeking care, public hospitals and clinics rarely need to promote their services. In contrast, private facilities – most of which are newly founded – need to advertise to attract patients. In addition to word-of-mouth, many marketing activities are implemented by private providers such as mass media promotion, including newspaper advertisement, television, radio, printed pamphlets, open seminars, and workshops to increase the spread of information about their services. According to many interviewees, this has been effective in attracting patients and is sometimes crucial for the existence of private health businesses, as stated in the following quotes:

Private providers can do very well in communication and marketing, they even have the art of attracting customers. I say it directly and frankly. The view that patients are not customers is pretty old, nowadays patients are customers, so private facilities have to lure guests with a lot of art. In that sense, the private facilities definitely are much better than the public ones. (Participant 17, Private Medical Association specialist).

Our hospital is newly founded. Some patients come to this hospital because of marketing programs and public events. Within the last two months, we have organised two domestic events and one international seminar, and thanks to that, a lot more patients [have] come to us. (Participant 11, director of private hospital_2)

Similar comments were offered by private clinic owners:

Even large private hospitals could fall and collapse if they do not have appropriate advertising strategies. So do private clinics. That is to say, the market eliminates those not only with low expertise but also with low advertising skills and customer care approaches. (Participant 13, private clinic owner_3)

We advertise by distributing flyers in surrounding areas where we think our clinic can attract people’s attention. It is fairly effective. (Participant 18, private clinic owner_4)

Many private providers alluded to creative healthcare advertising programs for the wider community, especially disadvantaged populations, as evident at the following private hospital:

We usually broadcast health education programs on TV. In addition, we usually participate in charity programs, providing free medical treatments for ethnic minorities or people living in very difficult areas, for example, places hardest hit by floods. (Participant 24, director of a private hospital_3)

However, some participants raised serious concerns about overreach and misinformation in advertising that could mislead patients. Several interviewees raised these concerns, as shown in the quote below:

The most important thing (that makes people use private health services) I think is that private providers are very strong in public relations and in the advertisement. I do know some private clinics, their medical quality is not so excellent, but they advertise very widely and loudly, so many people are attracted to that. (Participant 10, director of a public clinic)

A head of the Quality Assurance Department of a major public hospital acknowledged that marketing activities are somewhat excessive in relation to private providers:

One of the reasons people use private services is that private hospitals are doing communication and marketing much better than public hospitals. They even have staff waiting at the train stations, and bus stops to explain and pick up patients. (Participant 25, Head of the Quality Assurance Department of a major public hospital)

## Discussion

This qualitative study revealed that multiple social factors influence patients’ choice of private over public services in Vietnam, including word-of-mouth, the patient-doctor relationship and relationships among doctors, staff attitude and behaviour and marketing. Patients often base their healthcare choices on a variety of social channels to determine the credibility and trustworthiness of health providers. Private providers are familiar with and pay great attention to marketing to promote and persuade patients to use their services. At the present time, most public providers seem to ignore or show only limited interest in using different forms of social interaction to improve services to meet patients’ needs, especially those needs above and beyond strictly medical intervention. The importance, implications and broader bodies of research regarding these social concepts are explored below.

Silverman (2001) highlighted the strength of word-of-mouth as “the best way to make the decisions easier” as it is “a thousand times as powerful as conventional marketing”. In the digital era, this communication strongly influences the choice of medical services because it disseminates information rapidly without geographical and social barriers [7, 19, 20]. Word-of-mouth is also powerful because it connects with patients’ emotions and feelings, many of whom have serious diseases such as cancer, heart disease or HIV-AIDS [21]. Many private providers in Vietnam are utilising word-of-mouth as an effective and low-priced marketing tool. Their strategy is to satisfy patients’ needs as completely as possible so that they recommend the services to other people in the community. Unfortunately, public providers in Vietnam do not seem to harness the power of word-of-mouth to promote their service quality. This is one of their weaknesses that needs to be improved in the future if they are to maintain their reputation, market position and ability to compete for loyalty and trust in the community.

This study also found that, according to healthcare providers with extensive experience, many patients prefer to see public physicians they know well in their own private clinics. This is especially so when the doctor-patient relationship is positive and respectful. This is consistent with prior studies on the impact of the doctor-patient relationship on service options [22]. It is also clear from this study that relationships among doctors are influential because private doctors often refer patients to physicians in their personal, professional, and corporate networks. Patient referral is a daily currency in medicine due to patients’ complex medical needs, the clinical scope of practice of physicians and specialists, and the technical capacity of healthcare facilities. The referral is well regulated in the public system thanks to the clear referral networks to move patients from lower to higher level healthcare facilities. However, it could become unethical and even illegal if doctors referred patients for unnecessary diagnostic scans and treatments in order to receive high commissions and informal kickbacks rather than providing the best, evidence-based healthcare [23, 24]. These issues need to be regulated by the government to handle conflicts of interest and to protect patients from being treated as a commodity, especially in the Vietnamese context, where public doctors are lawfully investing, opening, and working in private facilities. Conflicts of interest could arise as patients are the source of income for those facilities.

Another finding is that staff attitudes and behaviour sometimes create serious problems in the public sector that damage the long-established good reputation of the public sector in Vietnam. Rudeness, arrogance and lack of empathy are often the subjects of complaints by consumers [25, 26]. The rudeness and unethical behaviour/attitude of some staff [27] are one of the reasons why many Vietnamese people do not seek care from public providers and prefer to pay out-of-pocket for private services where they get more personal care, even when treated by the same public doctors. Although the effect of poor staff behaviour on health outcomes sits outside of this study’s scope, this has been found to be detrimental in many contexts [28, 29].

On the other hand, the private sector has comparative advantages. Staff working in the private sector generally have much higher salaries, less workload per day, and better physical environments and staff morale than their colleagues in the public sector [30], and these characteristics give a competitive advantage over public providers. In addition, the support staff (receptionist, security, ushers) are well-trained to be enthusiastic and sensitive to patients’ needs. This would, in many cases, impress and satisfy patients even more than doctors’ medical care [9]. In addition, patients do not have to pay any bribery money and their payments for services are well explained by the staff. The advantages of the private sector provide a strong message to the public sector in regard to opportunities for change and improvement.

Internationally, the effect of competition among private and public providers on the quality and cost of services are controversial in many studies [31, 32]. Our study reveals public providers are well aware of their weaknesses, and in recent years have been seeking solutions to bridge the gaps with the private sector. This provides a strong signal that competition between private and public providers should improve, across the entire health system, the attitudes and behaviour of staff that could ultimately engender positive health outcomes and better overall experience for patients.

Finally, marketing is an aspect of free enterprise that is still relatively underdeveloped in the health sector within the socio-economic and historical context of Vietnam. While public health institutions currently do little to promote themselves, given their pre-eminent position in the health system, most private providers must use marketing to present their health services to consumers. The advertising in health care needs to be ethically regulated because it can mislead consumers [33, 34]. At present, healthcare marketing by private health services is not strongly regulated in Vietnam. Our study finds that some private clinics tend to provide inaccurate information on the expertise of the doctors working there to attract patients to their clinics. This over-advertising should be banned or well regulated to avoid consumers from unnecessary treatments, cost and potential harm.

Our study has several limitations. Firstly, although the analysis identified various problems in private health services such as over-pricing, over-servicing, and over-advertising, it may not fully reflect all problems and issues that could have been raised. Furthermore, it is not possible to assess how serious these problems are in relation to the veracity of observations about health service quality and patients’ safety in Vietnam. Another constraint is that the pool of key informants might not have been diverse enough to be representative of the views of actors throughout the health system.

## Conclusion

This study has implications for policy to change public services in Vietnam. It is recommended that public providers embrace social interaction with consumers as an effective strategy to improve their service quality. However, a cautionary note is that over-advertisement, over-servicing, and excessive focus on patients’ demands rather than medical needs, as well as the technical quality and safety of private providers, need to be appropriately regulated to protect patients from unnecessary treatments, costs and potential for harm. The insights from this study have direct relevance to many developing countries facing a similar challenge of appropriately managing the growth of the private health sector.

### Electronic supplementary material

Below is the link to the electronic supplementary material.

Supplementary Material 1

## Data Availability

The datasets used and/or analysed during current study available from corresponding author on reasonable request.

## Declarations

### Anonymity

All study participant names were coded and anonymised during data collection, data analysis and data presentation. Personal data on participants such as their official position and specific experiences were anonymised.
