# Supplementary material for: Why do consumers choose private over public health services? Reflective accounts of health providers in Vietnam
Source: BMC Health Serv Res. 2023 Aug 23;23:900. doi: 10.1186/s12913-023-09892-9 (PMC10464385; doi:10.1186/s12913-023-09892-9)
Supplement: Supplementary file 1 — Supplementary Material 1 [file 12913_2023_9892_MOESM1_ESM.docx]

**Appendices**

Table 1. Semi-structured interview respondents

| **No** | **Stakeholders in the health sector** | **Representing organisations** | **Number of informants** |
| --- | --- | --- | --- |
| 1 | National Assembly | Social affair committee | 1 |
| 2 | Government policy makers | Ministry of Health:  1) Department of finance and planning,  2) Department of medical services  3) Department of health insurance | 1  1  1 |
|  |  | Ministry of Planning and Investment | 1 |
|  |  | Hanoi department of Planning and Investment | 1 |
| 3 | Regulatory bodies | 1)Licensing and Accreditation-MOH  2) Social Health Insurance-MOLISA  3) Inspection-MOH  4) Quality assurance and management-MOH | 1  1  1  1 |
| 4 | Research institutions | 1) Hanoi Medical University,  2) Institute of Strategy and Health Policy | 1  1 |
| 5 | Professional networks | 1) Health Economic Association | 1 |
|  |  | 2) Private Hospital Associations | 1 |
|  |  | 3) Vietnam Private Medical Association | 1 |
| 6 | International organisations | World Health Organization | 1 |
|  |  | World Bank | 1 |
| 7 | Public providers | Public hospital leaders/managers | 2 |
|  |  | Public clinic leader(s) | 1 |
| 8 | Private providers | Private hospital leaders | 3 |
|  |  | Private clinics leaders | 6 |
| 9 | Joint-stock venture hospital | Department of internal medicine | 1 |
|  | **Total** |  | **30** |
